# Zika virus damages the human placental barrier and presents marked fetal neurotropism

**DOI:** 10.1590/0074-02760160085

**Published:** 2016-05

**Authors:** Lucia de Noronha, Camila Zanluca, Marina Luize Viola Azevedo, Kleber Giovanni Luz, Claudia Nunes Duarte dos Santos

**Affiliations:** 1 Pontifícia Universidade Católica do Paraná, Curitiba, PR, Brasil; 2 Fundação Oswaldo Cruz, Instituto Carlos Chagas, Laboratório de Virologia Molecular, Curitiba, PR, Brasil; 3 Universidade Federal do Rio Grande do Norte, Instituto de Medicina Tropical, Natal, RN, Brasil

**Keywords:** Zika virus, transplacental transmission, Hofbauer cells, neurotropism

## Abstract

An unusually high incidence of microcephaly in newborns has recently been observed in Brazil. There is a temporal association between the increase in cases of microcephaly and the Zika virus (ZIKV) epidemic. Viral RNA has been detected in amniotic fluid samples, placental tissues and newborn and fetal brain tissues. However, much remains to be determined concerning the association between ZIKV infection and fetal malformations. In this study, we provide evidence of the transplacental transmission of ZIKV through the detection of viral proteins and viral RNA in placental tissue samples from expectant mothers infected at different stages of gestation. We observed chronic placentitis (TORCH type) with viral protein detection by immunohistochemistry in Hofbauer cells and some histiocytes in the intervillous spaces. We also demonstrated the neurotropism of the virus via the detection of viral proteins in glial cells and in some endothelial cells and the observation of scattered foci of microcalcifications in the brain tissues. Lesions were mainly located in the white matter. ZIKV RNA was also detected in these tissues by real-time-polymerase chain reaction. We believe that these findings will contribute to the body of knowledge of the mechanisms of ZIKV transmission, interactions between the virus and host cells and viral tropism.

Zika virus (ZIKV) is an emerging flavivirus that belongs to the same family as the dengue (DENV), West Nile and yellow fever (YF) viruses (Pierson & Diamond 2013). From the time it was discovered in 1947, ZIKV has been associated with sporadic human infections in Africa and Asia (Dick et al. 1952, Hayes 2009). However, since 2007, the virus has been associated with large human outbreaks and a change in the pattern of the infections has been observed. High rates of infection and severe presentations, including neurological complications (*Guillain Barré* syndrome, meningoencephalitis), have been reported (Ioos et al. 2014, Oehler et al. 2014).

In early 2015, several patients presenting with dengue-like symptoms, such as mild fever, rash, conjunctivitis, and arthralgia, caught the attention of infectious disease physicians in the Northeast Region of Brazil. Although all patients lived in a dengue endemic area, dengue diagnosis was negative. Reverse transcriptase-polymerase chain reaction (RT-PCR) results from patients’ sera revealed autochthonous ZIKV infection in the country for the first time (Zanluca et al. 2015). Currently, more than 1,500,000 cases are estimated to exist in Brazil and ZIKV has spread to other South and Central American countries (PAHO 2016).

From October 2015 onward, an unusually high incidence of microcephaly in newborns was observed. Most of the women who delivered these children presented ZIKV-compatible symptoms during the first months of pregnancy. By February 2016, more than 5,600 suspected cases of microcephaly in newborns had been reported, representing a more than twenty-fold increase compared to the historical average of the last five years, and 120 suspected deaths due to microcephaly related to ZIKV have been reported to the Brazilian health authorities (EBC 2016). Other frequent causes of birth malformations, such as common viral infections and other infections, drug and alcohol abuse, preexisting disease, and genetic history, have been excluded. Moreover, ZIKV RNA was detected in amniotic fluid samples of two pregnant women who had ZIKV disease symptoms and whose fetuses were diagnosed with microcephaly (Calvet et al. 2016, Melo et al. 2016). Furthermore, viral RNA and protein were detected in newborn/fetal brain and placental tissues, which highlights the link between ZIKV infection in mothers and microcephaly in newborns (Martines et al. 2016, Mlakar et al. 2016). In this study, we describe ZIKV infection by anatomopathological, immunohistochemistry (IHC), real-time RT-PCR analysis and serological assays in placental tissues from women infected at different gestational time points (including first and third trimester of pregnancy) and in necropsy brain tissues from fetuses and newborns that died just after birth due to severe neurological disorders. These findings might contribute to the body of knowledge about the transplacental transmission and the neurotropism of ZIKV.

## MATERIALS AND METHODS

*IHC* - Formalin-fixed paraffin-embedded (FF-PE) tissue samples were stained using a conventional hematoxylin-eosin (H&E) technique (Baurakiades et al. 2011).

Sections from the blocks were analysed by IHC as described by Chong et al. (2009) with some modifications. Antigen retrieval was performed using the BioSB^®TM^ immunoretriever (Santa Bárbara, USA). The flavivirus-specific monoclonal antibody (MAb) 4G2 (hybridoma D1-4G2-4-15, ATCC HB-112) was used as a primary antibody, followed by the secondary antibody (Reveal Polyvalent HRP-DAB Detection System, Spring Bioscience) with a 30 min incubation at room temperature. The specificity of IHC staining was confirmed by omitting the primary MAb or using the non-related anti-Chikungunya virus MAb [named 1G1, produced at Carlos Chagas Institute (ICC-Fiocruz, Paraná, Brazil)]. The immunostained slides were observed using an optical microscope (Olympus™ BX50, Tokyo, Japan). For each sample, photomicrographs were taken in a high power field = 400x) using a Zeiss Axio Scan.Z1^TM^ scanner.

*RNA extraction and real-time RT-PCR* - To confirm the identity of the flavivirus in the IHC assays, the corresponding FF-PE tissue block was punched with a hollow needle, and tissue cores 3 mm in width were removed for molecular studies. Total RNA was extracted from these cores using the ReliaPrep^TM^ FF-PE total RNA Miniprep System (Promega) according to the manufacturer’s recommendations. When RNA was extracted directly from the *in natura* tissue (case 5), the RNeasy mini kit (Qiagen) was used following the manufacturer’s instructions. RNA was eluted in 50 µL of elution buffer, and 5 µL of the extracted RNA was amplified by real-time RT-PCR using two primer/probe sets specific for ZIKV (Lanciotti et al. 2008). Real-time assays were performed using the GoTaq Probe 1-Step RT-qPCR System (Promega) or the SuperScript III Platinum One-Step qRT-PCR System (Invitrogen) with amplification in the LightCycler 4800 instrument (Roche).

The amplification runs contained two negative and two positive controls. The negative controls consisted of a blank reagent with water and a negative human serum sample. For the positive controls, RNA extracted from a virus stock or from acute ZIKV human serum samples were used. The same tissue samples were also tested for the presence of DENV, another flavivirus endemic in the Northeast Region of Brazil. Real-time RT-PCR was performed using a published method for the detection of DENV-1, 2, 3 (Poersch et al. 2005) and/or an unpublished method for the detection of the four DENV serotypes (primer sequences available upon request). RNA extracted from the four DENV-serotype virus stocks were used as positive controls and a blank reagent with water and a negative human serum sample as negative controls.

To confirm the identity of the ZIKV in case 1, the amplicon was cloned in a pGEM-T Easy vector (Promega). Nucleotide sequencing was performed by the Macrogen Sequencing Service (Seoul, South Korea) with upstream and downstream primers of the cloning site.

*IgM antibody capture ELISA* - IgM antibodies were detected by an *in house* IgM capture enzyme-linked immunosorbent assay using inactivated cell-culture derived ZIKV and MOCK (from non-infected cell culture) as antigens, which were kindly provided by the Centers for Disease Control and Prevention. The ELISA was performed as described by Martin et al. (2000) with minor modifications. The positive samples were also tested with a commercial dengue IgM capture ELISA (PanBio) following the manufacturer’s instructions.

*Case reports* - The Molecular Virology Laboratory of ICC is one of the five official sentinel laboratories assigned by the Brazilian Ministry of Health to perform ZIKV diagnosis. In this context, we describe five cases of miscarriage, newborns with microcephaly or ZIKV infection during pregnancy.

As the samples were received for diagnosis, the patients’ clinical records were simplified. Even so, the laboratory analysis revealed relevant information about ZIKV infection and contributed to the knowledge about potential mechanisms of transmission and ZIKV neurotropism.

This work was approved by the Institutional Review Board (number 42481115.7.0000.5248).

*Case 1* - A 31-year-old woman living in the Northeast Region of Brazil confirmed her first pregnancy in May 2015. On May 8th, a first fetal ultrasound examination was performed and the results indicated a fetal size compatible with six weeks of development and normal heartbeats. On May 15th (seventh week of pregnancy), she presented fever and rash limited to two days. These symptoms combined with the ZIKV epidemic in the region led to a clinical-epidemiological diagnosis compatible with ZIKV disease. On June 19th (twelfth week of pregnancy), a second ultrasound examination failed to detect embryonic heartbeats and showed an embryo size compatible with eight weeks of development (embryo CRL of 17 mm). Curettage was performed on June 20th, and the placental tissue was fixed and embedded in paraffin for IHC and viral real-time RT-PCR analysis. Four FF-PE placenta tissue samples were received by the Molecular Virology Laboratory of ICC to diagnose ZIKV infection.

The patient indicated that she had a DENV infection some years ago and received the 17DD YF virus vaccine in 2013. Congenital infections with other agents, including Toxoplasma, *Treponema pallidum*, Rubella virus, Cytomegalovirus and Herpes simplex virus were negative according to appropriate serological assays. There were no records of drug or alcohol abuse, co-morbidities or genetic background that could be related to the abortion.

*Case 2* - A female with microcephaly and malformations of the feet and hands was born at 38.4 weeks of gestation by vaginal birth and died within 6 h. It was reported that the mother had a rash about a month before pregnancy and the doctor suspected viral infection; however, no laboratory diagnosis was performed. The mother was a 19-year-old woman that lived in the Northeast Region of Brazil. This was her second pregnancy and the first baby was born without any malformations. There was no kinship between the parents. There are no records of any contact with chemicals.

*Case 3* - A newborn male was born at nine months of gestation by cesarean delivery and died within 20 h. It was reported that the mother had a viral infection (not laboratory confirmed) in the third month of pregnancy. Also in the third month of pregnancy, ultrasonography revealed fetal microcephaly and malformations of the limbs and genitalia. The mother was a 26-year-old woman that lived in Northeastern Brazil. This was her first pregnancy and she denied the use of medication during the pregnancy.

*Case 4* - A newborn male was born at 35 weeks of gestation with a head circumference of 29 cm and died the day after birth. The microcephaly was detected postpartum. Serum and brain, liver, spleen, heart, adrenal gland and lung tissue samples were received. The mother was a 21-year-old woman that lived in Southern Brazil.

*Case 5* - A 31-year-old woman living in the Southern Region of Brazil had an exanthematic illness during the eighth month of pregnancy. Serum and urine samples were collected five days after the onset of symptoms for laboratory diagnosis. Five weeks later, the baby was born without microcephaly and without other apparent complications. Placenta fragments, umbilical cord blood and newborn serum samples were received for analysis. Tests for infection with other agents, including Toxoplasma, *Treponema pallidum*, Cytomegalovirus, HIV, and Hepatitis B, were negative.

## RESULTS

*Case 1* - The FF-PE placenta tissue samples (from the retained abortion) included the decidua, amnion and placenta. Slides stained with H&E showed chronic placentitis (TORCH type) with chronic villous inflammation (histiocytic-predominant villitis), edema and trophoblastic epithelium lesions when compared with normal villous tissue. There was a hyperplasia in villous Hofbauer cells, villous stromal lymphocytic cells and some histiocytes in the intervillous spaces. There were no significant histological findings to confirm chronic chorioamnionitis and deciduitis in samples of this study. There were no fetal samples available in all sections analysed.

**Fig. 1:**
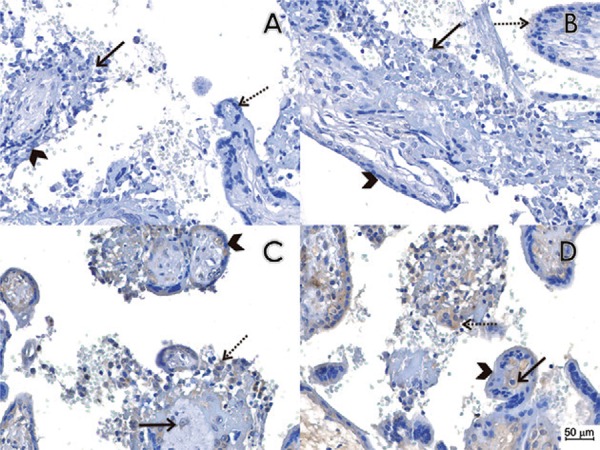
: pathological findings and immunohistochemistry reactions in placental tissues. (A) Histological section of case 1 immunostained by the conventional immunohistochemistry technique, omitting the primary antibody, which was used as a negative control. We observed chronic placentitis (TORCH type) with chronic villous inflammation (histiocytic-predominant villitis - arrow), edema and trophoblastic epithelial lesions (arrow head) as compared to normal villous tissue (dashed arrow). There was an increase in villous Hofbauer cells and villous stromal lymphohistiocytic cells. (B) Histological section of case 1 immunostained with a non-related anti-Chikungunya virus monoclonal antibody as the primary antibody, which was used as a negative control. We observed the same features observed in A (arrow shows histiocytic-predominant villitis, arrow head shows non lesional trophoblastic epithelial cells and dashed arrow shows normal villi). (C-D) Histological section of case 1 immunostained with the anti-flavivirus monoclonal antibody 4G2. Chronic placentitis (TORCH-type) was observed with immunopositivity in Hofbauer cells (arrow) and some histiocytes in the intervillous spaces (dashed arrow). There was no immunopositivity in the trophoblastic epithelium (arrow head).

IHC analysis with the anti-flavivirus monoclonal antibody 4G2 showed immunopositivity in Hofbauer cells and some histiocytes in intervillous spaces. There was no immunopositivity in the trophoblastic epithelium (Fig. 1C-D). To confirm the identity of the virus, RNA was extracted from two cores of the FF-PE tissue block corresponding to the areas of villitis. The specimens were positive in the two real-time RT-PCR amplification assays, with a threshold cycle lower than 34. In addition, RT-PCR tested negative for DENV.

**Fig. 2:**
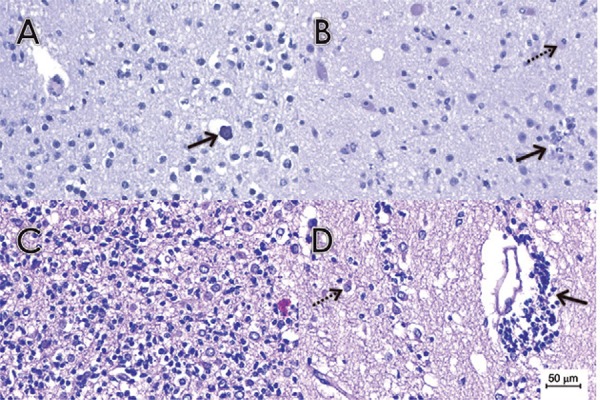
: anatomopathological findings on brain tissues samples stained with H&E. (A) Histological section of brain tissue samples of case 2 with a mildly affected white matter region revealing diffuse microglial hyperplasia, gliosis with reactive astrocytes and microdeposits of calcium (arrow). (B) Histological section of a brain tissue sample from case 3 with a mildly affected white matter region containing microglial nodules (arrow) and neuronophagia (dashed arrow). (C) Histological section of a brain tissue sample from case 4 with a more severely affected white matter region, revealing extensive destruction and infiltration by mononuclear inflammatory cells. Diffuse microglial hyperplasia was also present. In addition, severe gliosis with reactive gemistocytic astrocytes were diffusely distributed. (D) Histological section of a brain tissue sample from case 4 with a more severely affected white matter region showing extensive perivascular cuffing by lymphocytes (arrow). In addition, moderate gliosis with reactive gemistocytic astrocytes (dashed arrow) were diffusely distributed.

**Fig. 3:**
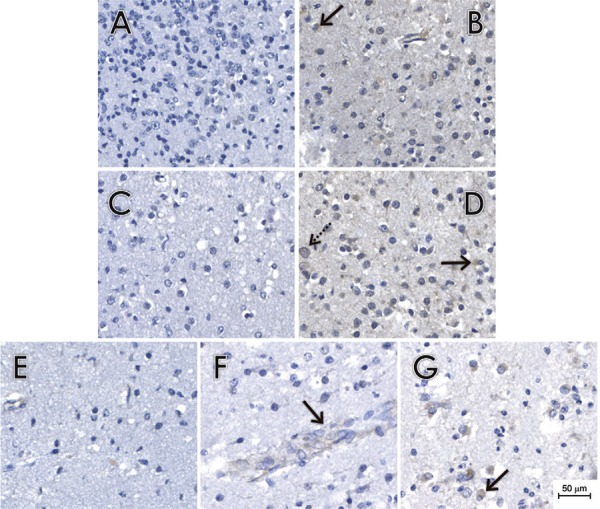
: pathological findings and immunohistochemical reactions in brain tissues. (A-D) Histological sections of brain tissue samples of cases 2 (A-B) and 3 (C-D) immunostained with 4G2 (B and D) or with a non-related anti-Chikungunya virus monoclonal antibody (A and C). We observed some positive glial cells (arrow) and scattered foci of microcalcifications (dashed arrows). (E-G) Histological sections of brain tissue samples of case 4 immunostained with 4G2 (F-G) or with a non-related anti-Chikungunya virus monoclonal antibody (E) as the primary antibody. We observed some positive gemistocytic glial cells (G - arrow). We also observed scattered positive endothelial cells (F - arrow).

*Case 2* - H&E slides from the brain, liver, lung, kidney, spleen and placenta showed several pathological alterations. Histology of brain tissue revealed no infiltration of leptomeningeal lymphocytes and no other pathological changes were observed except for mild vascular congestion. Mildly affected gray and white matter regions were observed to contain perivascular cuffs of mononuclear inflammatory cells, especially lymphocytes and microglial nodules, often surrounding degenerating neuronal cell bodies (neuronophagia). More severe affected gray and white matter regions revealed extensive destruction and infiltration by mononuclear inflammatory cells, perivascular cuffing by lymphocytes and clusters of microglia and macrophages marking sites of neuronophagy. Diffuse microglial hyperplasia was also present in all brain tissue samples. In addition, severe gliosis with reaction gemistocytic astrocytes and microdeposits of calcium are also diffusely distributed (Fig. 2A). IHC with the 4G2 anti-flavivirus monoclonal antibody analysis showed diffusely distributed immunopositivity in some glial cells (Fig. 3A-B).

Liver tissue sections showed moderate extramedullary hematopoiesis, slightly higher than normal levels of lymphohistiocytic cells in periportal spaces and mild vascular congestion (Supplementary figure D).

Lung tissues presented moderate vascular congestion, edema and some vacuolated alveolar macrophages (Supplementary figure A).

H&E results showed that the kidney and spleen displayed moderate vascular congestion. No other pathological changes were observed.

Samples from the placenta exhibited a villous structure, chorionic amnion and decidua. No umbilical cord samples were analysed. The villous structure of the placenta showed distal villous hyperplasia (villous immaturity or delayed villous maturation). Persistence of the cytotrophoblastic layer and thickening of the trophoblastic basement membrane were present. There was some degree of villous hypervascularity and stromal fibrosis. Several terminal villi seemed to be edematous, with reduced numbers of syncytial knots and slightly higher than normal levels of lymphoplasmacytic cells. In addition, Hofbauer cell hyperplasia with diffusely distributed focal villous sclerosis with focal calcifications and moderate increases of intravillous and perivillous fibrinoid deposits were observed. Vascular changes in villous stems were detected as hyperplasia of the muscular layer associated with stromal fibrosis. Moreover, mild chronic lymphocytic deciduitis of the decidua basalis was also observed. There was no chorioamnionitis in these samples (Supplementary figure F).

There was no reliable immunopositivity in the lung, liver, spleen and kidney. Real-time RT-PCR analysis detected the presence of ZIKV in placental and brain tissues punched from specific damaged tissue areas, with a negative result for DENV. ZIKV RNA was not detected in liver tissue; RT-PCR was not performed in the other tissues, since they were all negative in the IHC analysis.

*Case 3* - H&E slides from the brain, liver, spleen, heart and kidney showed remarkable anatomopathological injuries. Histology of the brain, liver and spleen revealed similar pathological alterations as those of case 2 (Fig. 2B, Supplementary figure E).

Heart tissue samples demonstrated mild vascular congestion. In H&E stained kidney samples, patchy distributed sclerosis of the glomeruli and moderate vascular congestion were observed (Supplementary figure E).

IHC analysis revealed diffusely distributed immunopositivity in glial cells (Fig. 3C-D). No reliable immunopositivity was observed in the heart, liver, spleen and kidney tissue samples. Real-time RT-PCR assays yielded positive results for ZIKV RNA in brain tissue samples and negative results for DENV RNA.

*Case 4* - H&E slides from the brain, liver, spleen, heart, adrenal gland and lung displayed several anatomic abnormalities. Histology of the brain, spleen and heart samples revealed similar pathological changes as those observed in cases 2 and 3 (Fig. 2C-D). Adrenal gland samples showed no pathological alterations but severe vascular congestion. In H&E stained liver sections, severe extramedullary hematopoiesis, slightly higher than normal levels of lymphohistiocytic cells in periportal spaces and severe vascular congestion were observed. Moderate hypoxic-type steatosis was also present (Supplementary figure C). Lung tissue presented severe vascular congestion and edema, diffusely distributed hyaline membranes (respiratory distress of prematurity) and vascular changes suggestive of persistent fetal circulation (hyperplasia of muscular layer of alveolar artery) (Supplementary figure B).

The IHC analysis showed diffusely distributed immunopositivity in glial cells (Fig. 3E-G). No reliable immunopositivity in the heart, liver, spleen, adrenal gland or lung was observed.

*Case 5* - In this case, all the specimens were tested by real-time RT-PCR, and samples of sera and umbilical cord blood were also tested by ELISA. The analysis of serum and urine samples from the mother, collected five days after the onset of symptoms (during the third trimester of pregnancy), revealed the presence of ZIKV RNA in the urine sample and anti-ZIKV IgM in the serum sample, confirming acute ZIKV disease. DENV infection was excluded by both assays.

In the analysis of the samples collected after delivery (placenta tissue, umbilical cord blood and newborn serum samples), viral RNA was isolated from placenta tissue. RT-PCR was negative for DENV. As the samples were received frozen, it was not possible to performed IHC analysis. Umbilical cord blood and the newborn serum samples yielded negative results for the presence of ZIKV RNA and anti-ZIKV IgM.

## DISCUSSION

In this study, we present new evidence of the harmfulness of ZIKV infection. Samples from a miscarriage at eight weeks of gestation (embryo stage), which occurred one week after the supposed ZIKV infection, exhibited chronic placentitis. Chronic inflammatory lesions of the placenta are characterised by the infiltration of the organ by lymphocytes, plasma cells, and/or macrophages and may result from infections (chronic placentitis TORCH-type) or be of immune origin (maternal anti-fetal rejection) (Greenough 1994).

TORCH-type chronic placentitis is common in Brazil and in many other countries, but it is more often diagnosed in the last weeks of pregnancy (Greenough 1994, Baurakiades et al. 2011, Kim et al. 2015). In this study, we report TORCH-type chronic placentitis associated with ZIKV in a placenta from a twelve-week-old pregnancy.

Vertical transmission of flaviviruses has been described in West Nile virus, resulting in congenital chorioretinal scarring and central nervous system malformation (Alpert et al. 2003). In addition, indirect evidence has demonstrated congenital YF vaccine virus after immunisation in pregnancy without any apparent involvement of the infant (Tsai et al. 1993).

Fetal infection is undoubtedly most often acquired during primary maternal infection by the passage of virions through the trophoblasts (Benirchke et al. 2006a). To our knowledge, this is the first report of TORCH-type chronic placentitis associated with ZIKV. Maternal cellular infiltration as well as an increase of Hofbauer cells (placental macrophages) are common features of TORCH-type chronic placentitis. The activation of macrophages in the villi has been implicated in the destruction of the villous architecture and the trophoblastic epithelium, and these findings are consistent with alterations in the placental immunological barrier. In the cases reported herein, viral protein were detected in IHC by MAb 4G2 in Hofbauer cells as well as maternal histiocytes. Hofbauer cells have the migratory ability to reach the fetal vessels and then infect the fetal cells (Baurakiades et al. 2011, Kim et al. 2015). A reasonable hypothesis would be that ZIKV might be using the Hofbauer cells and their migratory ability to reach the fetal vessels.

Although none of the cases reported here presented significant histological aspects to confirm chronic chorioamnionitis or deciduitis, three of them exhibited immature placentas (Benirchke et al. 2006b) and their newborns had congenital anomalies. There are no reports in the literature to date of placental immaturity with ZIKV infection and vertical transmission. The few reports describing maternal *Flaviviridae* infections and congenital transmission did not report on placental lesions (Boussemart et al. 2001, Alpert et al. 2003). There are recent reports of placental calcifications, fibrosis, fibrin deposition and villitis associated with maternal ZIKV infections, but none refer to immaturity (Martines et al. 2016, Mlakar et al. 2016).

We also demonstrated severe fetal brain injury associated with the vertical transmission of ZIKV. This adds ZIKV to the list of other flaviviruses known to cause encephalitis, such as West Nile and Japanese and St. Louis encephalitis (Love & Wiley 2002). Brain lesions from cases 2, 3 and 4 had anatomopathological characteristics similar to encephalitis caused by those flaviviruses. However, ZIKV cases presented additional particularities, such as severe brain damage with diffusely distributed lesions, while previous studies have demonstrated chronic leptomeningitis and brain lesions located in the cortical and subcortical white matter (Hollidge et al. 2010, Misra & Kalita 2010, Murray et al. 2010). Previous studies demonstrated the teratogenic effects of flaviviruses (Laszczyk et al. 2007) with neurotropism, including ZIKV, which has been associated with congenital brain anomalies (Martines et al. 2016, Mlakar et al. 2016, Sarno et al. 2016). The flavivirus envelope protein was present in brain tissues from newborns with microcephaly and ZIKV was confirmed by real-time RT-PCR of the tissue samples. The presence of the viral envelope protein was not observed in other organs, in accordance with previous findings on ZIKV infection during pregnancy (Martines et al. 2016, Mlakar et al. 2016, Sarno et al. 2016). This indicates that the brain was the main target organ for viral replication in the fetus, highlighting a strong neurotropism.

In addition, we described a case in which the ZIKV infection in the eighth month of pregnancy resulted in the infection of placental tissue, but not the fetus. In this case, ZIKV RNA was isolated from placental tissues, reinforcing the tropism of the virus by this organ and showing that it is independent of the gestation period. The absence of ZIKV RNA and anti-ZIKV IgM in the serum of the newborn strongly suggest that the fetus was not infected, although ZIKV had reached the placental tissues.

TORCH-type maternal infections, which are acquired earlier during pregnancy, may be associated with severe congenital anomalies. When these TORCH infections are acquired in more advanced stages of pregnancy (third trimester), infectious processes are often observed in the fetus, but congenital anomalies are less commonly observed (Baurakiades et al. 2011, Neu et al. 2015). A preliminary cohort study with pregnant women who presented febrile illness associated with rash performed in Rio de Janeiro, Brazil, found that the pathological changes during embryogenesis occurs predominantly in fetuses infected in the first trimester of development. On the other hand, central nervous systems abnormalities were also observed in some cases when the infection occurs in the third trimester of pregnancy (Brasil et al. 2016). A plausible reason is that fetal morphogenesis occurs in the first nine-12 weeks of gestation.

In the cases described here, among the expectant mothers that reported clinical ZIKV symptoms in the first trimester of pregnancy, one of them had villitis and exhibited early miscarriage, and the other two, although they did not have active villitis in the placenta, exhibited fetal brain abnormalities. On the other hand, in case 5, the expectant mother was infected in the third trimester of pregnancy and the placenta was positive for ZIKV, but the newborn did not have viremia or any congenital anomalies. In light of these observations, even considering the small number of cases, we presume that early fetal viremia, even in the embryonic period, could be more closely associated with congenital anomalies. Furthermore, pregnant women infected in the later stages of pregnancy may have sufficient placental maturity to avoid fetal contamination.

Our findings of placental inflammation and the presence of ZIKV in Hofbauer cells suggest that damage of the placental barrier may facilitate fetal infection, but this needs further confirmation. Similarly, the temporal persistence of ZIKV in placental tissues remains to be determined. A coordinated and extensive multidisciplinary research effort on the biology, transmission and interaction of ZIKV with the human host should be employed to address these issues.
